# Counting the Toll of Inflammation on Schizophrenia—A Potential Role for Toll-like Receptors

**DOI:** 10.3390/biom13081188

**Published:** 2023-07-30

**Authors:** Saahithh Redddi Patlola, Gary Donohoe, Declan P. McKernan

**Affiliations:** 1 Department of Pharmacology & Therapeutics, School of Medicine, University of Galway, H91 TK33 Galway, Ireland; s.patlola1@universityofgalway.ie; 2 School of Psychology, University of Galway, H91 TK33 Galway, Ireland; gary.donohoe@universityofgalway.ie

**Keywords:** inflammation, Toll-like receptor, schizophrenia, cognition

## Abstract

Toll-like receptors (TLRs) are a family of pattern recognition receptors (PRRs) that are ubiquitously expressed in the human body. They protect the brain and central nervous system from self and foreign antigens/pathogens. The immune response elicited by these receptors culminates in the release of cytokines, chemokines, and interferons causing an inflammatory response, which can be both beneficial and harmful to neurodevelopment. In addition, the detrimental effects of TLR activation have been implicated in multiple neurodegenerative diseases such as Alzheimer’s, multiple sclerosis, etc. Many studies also support the theory that cytokine imbalance may be involved in schizophrenia, and a vast amount of literature showcases the deleterious effects of this imbalance on cognitive performance in the human population. In this review, we examine the current literature on TLRs, their potential role in the pathogenesis of schizophrenia, factors affecting TLR activity that contribute towards the risk of schizophrenia, and lastly, the role of TLRs and their impact on cognitive performance in schizophrenia.

## 1. Introduction

Schizophrenia is a psychiatric disorder with a complex aetiology. Multiple genetic and environmental factors are associated with increased risk. We know from twin studies that an individual with a family history of schizophrenia is at increased risk [1]. Other contributing factors include exposure to adverse childhood experiences (e.g., physical/emotional neglect/abuse) and substance misuse during adolescence [2]. During pregnancy, exposure to infections, stress/trauma, or toxic materials by the foetus may also stimulate inflammatory responses that alter brain development [3,4]. Recently, there has been an increase in interest in the role of the immune system in the pathophysiology of both neurodegenerative (Alzheimer’s disease, Parkinson’s disease, multiple sclerosis) [5] and psychiatric disorders (schizophrenia, depression, and bipolar disorder) [6]. The concept of neuroinflammation in neurodevelopmental disorders is gaining wide interest, including, for example, the role of Toll-like receptors [7,8,9]. Toll-like receptors were initially identified in the developing embryo of *Drosophila melanogaster*, whose function was later discovered to be immune-related in humans, and played a crucial role in recognising invading pathogens [10,11]. 

Multiple theories have been proposed to understand variations in clinical symptom severity and cognitive deficits in schizophrenia. Immunochemical analysis (ELISA, multiplex, and fluorescent-based protein quantification assays) of blood plasma from schizophrenia patients shows alteration in the cytokine network, wherein elevation of proinflammatory cytokines was observed [12,13]. Positron emission tomography (PET) [14] and magnetic resonance imaging (MRI) [15] studies show microglial activation; a change in cortical thickness and grey matter volume is associated with elevated cytokine levels, indicating that cognitive deficits might be associated with an inflammatory response. Therefore, understanding the neuroinflammatory activity in schizophrenia is essential, and investigating TLRs could be pivotal in helping to clarify the mechanisms and pathways through which cognition is affected in schizophrenia.

The objective of this review is to highlight the current understanding of TLRs in the brain, their signalling, and their potential role in schizophrenia. We highlight the various factors involved in the risk of schizophrenia, and how TLRs are influenced by these factors and contribute to the risk of neuroinflammation. Finally, we showcase the current understanding of TLR involvement in cognitive deficits in schizophrenia, along with a possible pathway connecting the neuroinflammatory response to cognitive deficits. 

## 2. Toll-like Receptors

Toll-like receptors (TLRs) are mediators of the innate immune system, and respond to pathogens primarily through initiating inflammation. TLR genes were first located on human chromosomes in the late 1990s, and were found to be structurally similar to Toll in the common fruit fly (*D. melanogaster*) [16]. Toll-like receptor 4 (TLR4) was the first human TLR to be cloned, and later many others were identified [16,17]. At present, we have 10 known family members in humans, each capable of recognising distinct molecular (proteins/lipids) patterns [16,18,19,20]. These TLRs are transmembrane proteins present almost everywhere in the body, but are highly expressed by immune cells such as macrophages, dendritic cells, mast cells, etc. [20,21]. Most of these are present on the plasma membrane (TLR1, 2, 4, 5, 6, and 10) while the rest of them (TLR3, 7, 8, and 9) are inside the cells, on the endosomal membrane [21,22,23]. 

TLRs, along with nucleotide oligomerization domain (NOD)-like receptors (NLRs), C-type lectin receptors (CLRs), retinoic acid-inducible gene-I (RIG-I)-like receptors (RLRs), and absent in melanoma-2 (AIM2)-like receptors (ALRs) together belong to a receptor superfamily called pattern recognition receptors (PRRs) [24]. The name itself describes that they recognise specific molecular patterns on pathogens and initiate a cascade of signalling. The patterns recognised by TLRs belong to various pathogens such as bacteria, viruses, fungi, yeast, pollen, etc. In fact, they do not recognise these pathogens as a whole, but only specific molecular or genetic patterns of these pathogens. These specific proteins are called pathogen associated molecular patterns (PAMPs), such as lipoproteins, lipopolysaccharides, zymosan, flagella, and single/double stranded DNA and RNA [25,26,27]. In addition, TLRs also recognise molecules that are secreted internally as a consequence of internal damage, injury, stress, or repeated insult. These proteins are referred to as damage associated molecular patterns (DAMPs), and include heat-shock proteins, S100 family, HMGB1, hyaluronic acid, fibrinogen, microRNAs, etc. The interactions between these molecules and TLRs result in an inflammatory response mediated by cytokines, interferons, and chemokines [28,29]. 

**Figure 1:**
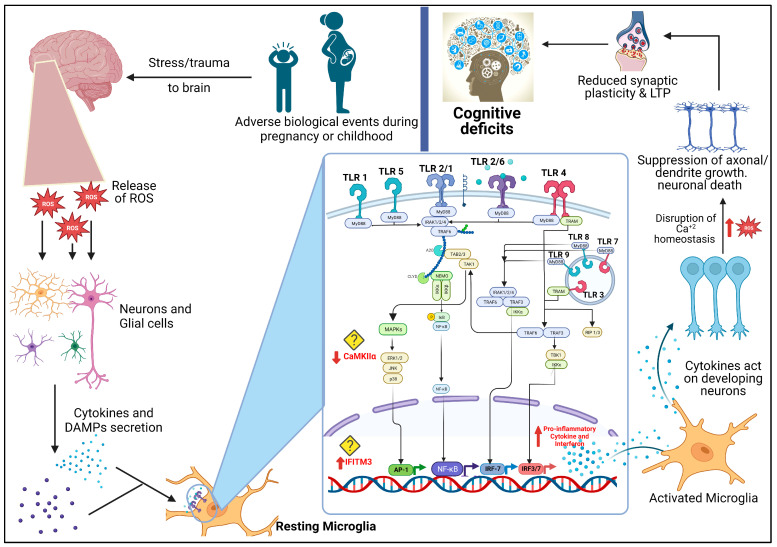
Figure illustrating the hypothesis of TLR pathway and signalling leading to cognitive deficits in patients suffering from schizophrenia. ROS—reactive oxygen species, DAMPs—damage associated molecular patterns, TLR—Toll-like receptor, LTP—long term potentiation, CaMKIIα—calcium-calmodulin dependent kinase II alpha, IFITM3—interferon-induced transmembrane protein 3, MyD88—myeloid differentiation primary response 88 protein, IRAK—interleukin-1 receptor-associated kinase, TRAF—tumour necrosis factor receptor (TNFR)-associated factor 6, TRIF—TIR-domain-containing adapter-inducing interferon-β, TRAM—TRIF-related adaptor molecules, TAK—transforming growth factor-β (TGF-β)-activated kinase 1, TAB—TAK1 binding protein 1, IKK—IκB kinase, IκB—inhibitor of nuclear factor kappa B (NF-κB), MAPK— Mitogen-activated protein kinase, ERK—extracellular signal-regulated protein kinase, JNK—c-Jun N-terminal kinase, RIP—receptor-interacting protein, IRF—interferon regulatory factor, AP-1—activator protein 1. “Created with BioRender.com (accessed on: 16 May 2023)”.

TLRs have an N-terminal domain present outside the cell membrane, and a Toll/IL-1 receptor (TIR) domain inside the cell membrane. PAMPs and DAMPs are recognised by the former domain, and downstream signalling is mediated by the latter [30,31]. Downstream signalling results in the production of inflammatory mediators such as cytokines, and chemokines such as interleukin 6 and tumour necrosis alpha. Proteins and pathways involved in this signalling differ among TLRs, and are also dependent on the type of stimuli. In the case of TLR2, it detects a multitude of ligands that are part of various pathogens: most Gram-positive and some Gram-negative bacteria, fungi, and host proteins [32]. Similarly, TLR4 recognises ligands from Gram-negative bacteria, whereas TLR3, 7, 8, and 9 recognise genetic material from viruses and bacteria. These TLRs also recognise endogenous compounds such as heat-shock proteins, S100 proteins, fragmented genetic components, etc. [33]. The response to these exogenous/endogenous ligands is exerted through TLR’s downstream signalling, which is broadly divided into two pathways (Figure 1): the MyD88 (myeloid differentiation primary response 88 protein) dependent pathway, and the TRIF (TIR-domain-containing adapter-inducing interferon-β) dependent pathway. All TLRs initiate the MyD88 pathway except TLR3, which initiates the TRIF pathway, whereas TLR4 is involved in both pathways. These pathways are discussed in detail somewhere else [30,34].

## 3. Toll-like Receptors in Central Nervous System (CNS) Immune Regulation

### 3.1. TLR Signalling in the Brain

The blood–brain barrier (BBB) is a multilayered membrane that selectively allows the entry of exo/endogenous compounds to the brain and restricts most other substances, including antibodies, immune cells, and mediators [35]. To facilitate immune function during adverse events, the CNS has its very own network of immune cells that includes non-neuronal cells such as microglia, oligodendrocytes, and astrocytes (one of the three layers of the BBB) that mediate the innate and adaptive immunity in the CNS [35,36]. These three cell types play an active role in the innate immune response mediated through TLRs. Expression of TLRs in the human CNS was first observed in postmortem brain sections of white matter tissue. Human adult tissue samples and mRNA analysis showed that brain cells expressed all 10 TLRs (1–10), with TLR4 showing the highest expression, followed by TLR2, 6, 7, 9, and 3 [37,38].

Studies conducted using primary cultures reported the expression of TLRs in individual cells. It was observed that all human glial cells expressed TLR2 and TLR3, wherein astrocytes expressed high TLR3 and oligodendrocytes showed higher TLR2; microglial cells express all subgroups of the TLR family [37]. Later studies showed that astrocytes also express the rest of TLRs [39]. Studies have confirmed the presence of multiple TLRs on neuronal cells: TLR1, 6 [40], TLR2, 4 [40,41], TLR3 [41,42], TLR7 [43], TLR8 [44], TLR9 [45]. These TLRs are found to be actively involved in the pathophysiology of conditions such as ischaemia, stroke, and viral infections, and interestingly, they are capable of eliciting immune responses independent of glial cells [41,42].

Despite the aforementioned evidence of TLR’s active role in the CNS, a human embryonic astrocyte culture study showed preferential TLR3 activity. Although the culture was stimulated with TLR2, 3, 4, 5, and 9 agonists, only TLR3-stimulated cells showed elevation in proinflammatory cytokines, while the others did not [46]. Similarly, in another study, astrocyte cultures showed no activation of TLR4 post-LPS (lipopolysaccharide) treatment (LPS is a TLR4 agonist) either by MyD88 or TRIF pathways, which is contrary to the response elicited by microglia [47]. This might be the case because in both studies, the primary astrocyte cultures used were of embryonic origin. Stimulation studies demonstrate that human microglial cells from foetal cerebral tissue [39] responded to ligands that activate TLR2, 3, and 4 by releasing cytokines such as interleukin-6 (IL-6) and tumour necrosis factor-α (TNF-α), whereas astrocytes elicited a response only to polyinosinic:polycytidylic acid (poly I:C) stimulation by significantly elevating IL-6, TNF-α, and chemokine expression, indicating the activation of TLR3. These studies indicate the limited role of astrocytes in innate immunity at embryonic stages [39,46].

### 3.2. Role of TLR in Neurodevelopment and Pathogenesis

TLRs play a crucial role in the protection of the central nervous system by mediating the innate immune response. Activation of each subtype of TLR depends on specific circumstances, such as, TLR2-mediated neuroinflammation led to increased permeability in the BBB, promoting bacterial clearance in mice during meningitis; the absence of TLR2 led to the disruption in the conventional (TLR2-TNF-α) inflammatory response, and ultimately caused detrimental effects to the CNS [48]. Similarly, in vitro studies demonstrated that TLR3 activation in response to viral infections showed neuroprotective activity facilitated by cytokines (IL-6, TNF-α), chemokines (CCL-5), and interferons (IFN-β). Furthermore, it was observed that potent viral infection triggers neuroprotective effect through inflammation via TLRs, but on the other hand, it had a detrimental effect on the CNS, as the neuroinflammation simultaneously increased the viral invasiveness [49]. Moreover, viral stimuli and higher immune activity can have a degrading effect on developing neurons and prevent axon and dendrite growth, further affecting synapse formations [50].

Similarly, TLR activation can have a deleterious effect on brain development in the foetus or later in life. It was observed in animals that in utero activation of TLR3 and 4 negatively affects foetal neurodevelopment. Poly I:C (a TLR3 agonist) treatment in animals during gestation caused defective development of cerebral cortical cells, followed by behavioural changes (decreased locomotor activity). The detrimental neurodevelopmental changes in the cells were mediated by TLR3, followed by the upregulation of multiple cytokines and chemokines [51,52]. Moreover, such activation changed the expression of proteins responsible for neurogenesis and plasticity, causing impaired synapses and long-term plasticity, which may have been the reason for the aforementioned behavioural changes [53,54].

Mouse studies also demonstrated changes in foetal neurogenesis after maternal immune activation (MIA) by LPS. These changes were mediated by TLR4, leading to the elevation of proinflammatory cytokines and an impaired foetal brain due to hypoxia [55]. It was observed that energy deprivation (normally observed in hypoxia) led to neuronal death mediated by TLR2- and 4-dependent caspase pathways [41]. Furthermore, in vitro studies illustrated a peculiar effect of TLR4 activation amongst the glial cells. LPS-induced activation of TLR4 in microglial cells was found to damage the developing oligodendrocytes, which potentially could cause neurodegeneration due to the lack of myelin sheath [56]. Another study utilising a sophisticated triculture system (endothelial cells, microglia, and neurons) showed an increase in BBB permeability caused by neuroinflammatory response (elevated IL-6, TNF-α, and nitric oxide) post-LPS treatment via TLR4/NF-kB signalling. Moreover, this neuroinflammation model explained neuronal injury through caspase-3 and Tau (proteins part of TLR4 downsignalling) [57]. Such external stimuli-induced neurodegenerative effects in developing neuronal cells could be applicable in the human MIA model by infections or diseases during foetal development.

## 4. The Role of Toll-like Receptors in Schizophrenia

Schizophrenia is a debilitating psychiatric disorder affecting 0.45% of adults worldwide, according to the World Health Organisation, [58]. According to 2019 data, the prevalence of schizophrenia ranges from 0.2 to 0.5% across nations worldwide [59]. Emerging evidence suggests the role of the immune system and its various components in the risk of schizophrenia, including cytokines, C-reactive protein, chemokines, antibodies, etc. [60]. Multiple studies show altered protein and mRNA expression of TLRs in schizophrenia (Table 1) compared to healthy adults [61,62,63]. Human postmortem studies carried out on individuals diagnosed with schizophrenia, brain samples showed lower *tlr4* mRNA and protein levels, and downregulation of *il6*, *il10*, and *tnfa* mRNA in the prefrontal cortex (PFC) region [64,65], and elevated TLR4 protein levels in the cerebellum region [64]. On the contrary, high *tlr4* and *myd88* mRNA expression was observed in the PFC region of the human brain [66]. On the flip side, studies on peripheral blood show downregulation of *tlr3* and *5* mRNA levels and upregulation of *il6* and *il10* mRNA levels in patients with schizophrenia [67], which is in contrast to evidence from brain samples. Studies of acute and first-episode psychosis (FEP) patient groups reported higher expression (measured by mean fluorescence intensity (MFI)) of TLR3, 4, and 5, but not TLR2, in monocytes [68,69]. Although Keri et al. observed higher TLR4 MFI in schizophrenia post-TLR4 stimulation, the MFI expression of IL-6, TNF-α, and IL-1β was lower than controls [69]. Similar results were also observed by Muller et al. with IL-1β. This was interpreted to represent an increase in TLR expression to compensate for the functional deficit [68]. In contrast, whole blood stimulation studies in schizophrenia showed elevated concentrations of IL-6, TNF-α, and IL-1β post-TLR2 stimulation, and increased IL-1β alone post-TLR4 and 8 stimulation [7]. Individual blood cell studies in FEP patients showed lower TLR protein expression in monocytes, B cells, and T cells [63]. Overall, we can infer that dysregulation of TLR expression exists in patients with schizophrenia and varies at various stages of disease progression, but we do not have sufficient evidence to implicate their extent of involvement in the immunopathology of schizophrenia.

The concept of immune-mediated risk for schizophrenia has existed since the late 1920s. The first instance of this was observed in the years following the Spanish flu pandemic, when some patients with influenza showed psychosis-like symptoms [73]. After this, the theory of virus-induced psychosis became popular (see [74] for more details), and many other infections from viruses, bacteria, and parasites were linked to the risk of schizophrenia, such as herpes simplex virus (HSV) [75], Epstein–Barr virus [76], and *Toxoplasma gondii* [77]. The most common theory about infections in relation to schizophrenia is related to the stimulation of the foetal immune system due to maternal infection, or by the antibodies generated by the mother in response to infection, ultimately posing a risk of schizophrenia in the offspring [78].

### 4.1. Maternal Immune Activation (MIA) and Early Life Exposure

Increased risk for psychosis-like symptoms following MIA (inflammatory stimuli due to TLR activation), conventionally associated with increased production of cytokines and chemokines, in offspring has been theorised for a long time [79,80]. Infections such as cytomegalovirus, influenza, herpes simplex virus, and coronavirus lead to upregulation of TLR mRNA (2–4 and 7–9). This is also accompanied by an increase in interferon and cytokine production causing complications in pregnancy, miscarriage, or foetal abnormalities [81,82]. These elevated cytokines and chemokines can migrate from the placenta into the foetus, and such exposure could cause changes to the CNS, leading to abnormal neurodevelopment [83]. Moreover, conditions such as diabetes mellitus, obesity, and systemic lupus erythematosus (SLE) in pregnancy were linked to high expression of TLRs, cytokines, and chemokines in peripheral blood, and similar levels were reflected in the placenta [84,85,86]. This suggests that conditions other than viral/bacterial stimuli could lead to an elevation in proinflammatory cytokines that could cross the placenta leading to prenatal immune activation increasing the risk of schizophrenia.

One of the earliest epidemiological studies linking infection with schizophrenia was in 1989, which was based on the data of subjects born during the epidemic period (1918 and 1957 influenza epidemic) reporting schizophrenia. A significant risk (1.4 risk ratio) of schizophrenia was observed in the subjects whose mothers were exposed to infection during 5–7 months of pregnancy compared to other months of pregnancy in the Scottish population. The same was not observed if all United Kingdom subjects were considered [87]. Similarly, another study conducted on patients diagnosed with schizophrenia showed that the risk of schizophrenia greatly increased in offspring with elevated maternal antibodies against type 2 HSV infection during pregnancy [75]. Despite the small sample size (*N* = 27/50 case/controls), it was reported that elevated maternal serum TNF-α during pregnancy had an association with the risk (8.5 odds ratio (OR)) of schizophrenia later in their offspring [88]. Animal models show that neural progenitor cells (NPCs) (NPCs undergo multiple changes to develop into neurons) expressed TLR3 and IRF3, and these cells responded to both in vitro and in vivo (dams) stimulation with poly I:C. Although conflicting results were observed from both models, in vivo results showed a significant decrease in cell proliferation post-treatment [89]. Similarly, irregular differentiation (high cholinergic neuronal differentiation and undifferentiated progenitors) and neurodevelopment were observed when forebrain from rat embryos were cultured with microglial conditioned medium stimulated by LPS [90]. Such evidence points to the detrimental neurodevelopmental effects on the foetus during pregnancy postexposure to infections or stimuli (TLR ligands), which could later affect the offspring.

For a long time, TLRs have been considered a target for vaccine adjuvants to increase the efficacy of the vaccine, where they act by low-level stimulation of the target TLRs and activate the interferon pathway [91]. It was observed in animals that vaccination for influenza promoted better neural development (indicated by increased cell proliferation, neural differentiation, and expression of brain-derived neurotrophic factor (BDNF) in pups) and provided protective function in the mother and offspring through TLR4 stimulation [92]. The aforementioned evidence indicates that the extent of activation of TLRs is determined by the severity of the infection in mothers, further affecting the neurodevelopment in the offspring while appropriate care such as vaccination, where the virus cannot replicate, can promote protective effects through TLRs on the offspring.

Animal models show a more detailed picture of MIA (Table 2). Intraperitoneal administration of Poly I:C and LPS in dams led to upregulation of TLR3 and 4 mRNA expression in the hippocampus tissue of the offspring. Moreover, this upregulation was associated with sociocognitive deficits and behaviour abnormalities in them [93]. In another study, post-LPS treatment in dams, mice showed upregulation of TLR2 and 4 mRNA in addition to COX-2 (cyclooxygenase), iNOS (inducible nitric oxide synthase), and CCL2 (chemokine C-C motif ligand) in the foetal stage, and later in adolescence. This was suggested to affect the microglial and astrocyte development in the amygdala region of the brain [94].

Similar to the MIA model, exposure to harmful environmental stimuli during childhood or early stages of life can affect neurodevelopment, cognitive functions, and behaviour [102,103]. Such dysfunction later in life contributes towards the risk of psychosis/schizophrenia. A meta-analysis on early exposure to CNS infections showed that childhood exposure to viral, but not bacterial, infections led to twice the risk of schizophrenia during adulthood [104]. It was also observed in neonatal mice studies that an increase in *tnfa* mRNA and impairment in behaviour and memory resulted after repeated exposure to poly I:C (TLR3 agonist) for 5 days. Additionally, disruption in glutamatergic neurons in the hippocampus (responsible for learning and memory) was observed [98]. When neonatal rats were administered poly I:C intraperitoneally, reduced white matter density and slow myelin development were observed. Moreover, 12 weeks post-treatment, elevation of *tnfa* and downregulation of *bdnf*, *arc* (activity regulated cytoskeleton associated protein), and *egr1* (early growth response 1) mRNA (associated with neurodevelopment) in the hippocampus was observed, resulting in poor memory, motor activity, and coordination [105,106]. One of the recent studies demonstrated that, in patients with schizophrenia having exposure to childhood trauma, IL-6 played a role in mediating the biological events leading to social cognitive deficits [107]. Similarly, IL-6 was reported to mediate the development of internalising symptoms (anxiety, depression, and social withdrawal) in children after experiencing an adverse event [108]. Interesting findings from Chase et al. showed an increased expression of *il6* mRNA in the schizophrenia group, without any significant changes in *tlr4* mRNA expression. Furthermore, there was a significant decrease in *ifng* and *irf1* mRNA expression [62]. This indicates that the increase in *il6* mRNA expression could be due to other TLRs or other PRRs. The downregulation of interferons was probably due to the TLR4/IRF1-dependent pathway, as observed in macrophages [109]. Another possibility could be that there was an increase in TLR activity without any change in its expression.

### 4.2. Factors Influencing TLRs in Schizophrenia

#### 4.2.1. Diet and Gut Microbiome

There are many diseases and disorders that are associated with improper diet. Studies showed that prenatal malnutrition during famine throughout the world in various eras increased the risk of nervous system-related disorders and mental illnesses such as schizophrenia, antisocial behaviours, other psychiatric disorders, and congenital anomalies such as neural tube defects [110]. Although it is unclear if this was due to the direct result of malnutrition (diet change) or other underlying causes such as stress, it was observed in Dutch and Chinese populations who experienced the ‘Dutch hunger of 1944–1945’ and ‘Great leap forward of 1958–1962’, respectively, that the risk of acquiring mental illness, especially schizophrenia, was two-fold higher in the populations born during these great famines [110,111].

Similar to how dietary deficits can be dangerous to mental health, an imbalanced diet lifestyle can affect the same. The nutritional psychiatry division of Harvard reports that the type of food can affect the inflammatory status and oxidative stress in the body. It was observed that the traditional diet from Asian and Mediterranean areas proved to have a 25–30% lower risk of depression when compared to the western diet [112]. Moreover, studies show that saturated fatty acids (SFAs) are capable of activating microglia and inducing proinflammatory cytokines, which is facilitated by TLR2 and 4 [113]. The mRNA levels of *il6*, *il1b*, and *tnfa* were found to be elevated post-SFA treatment in mouse cell culture, and their upregulated expression was similar to that of LPS stimulation [114]. Furthermore, in in vitro studies (mouse and human), TLR2- and 4-dependent activations were observed with SFAs. TLR2, 3, 5, and 9 activations by their respective agonists were attenuated by ω-3- and ω-6-polyunsaturated fatty acids (PUFAs), indicating that some fatty acids activate TLRs, and some others inhibit their activity [113,115,116].

A high-fat diet is closely related to obesity [117], and it was observed that a high-fat diet elevated proinflammatory cytokine expression via the TLR pathway (NF-kB activation), causing an inflammatory state which correlated with weight gain [118,119]. This high-fat diet can also dysregulate the gut microbiome [120], due to bacterial metabolites migrating to the brain and crossing the BBB [121], and inducing inflammatory responses in the brain (mentioned in Section 3.1). This further affects neurodevelopment (discussed in detail elsewhere [122,123]). The “leaky gut” MIA model suggests that metabolites from maternal microbiome dysbiosis can act as inflammatory stimuli on the foetus [123] and, in turn, potentially increase the risk of psychiatric disorders such as schizophrenia.

Evidence also suggests that the presence of unwanted or harmful microbes such as *Saccharomyces cerevisiae* (induced gastrointestinal inflammation) is associated with schizophrenia in both FEP and chronic patients [123,124]. Furthermore, schizophrenia was found to be associated with gastrointestinal dysfunction symptoms. The prevalence rate of such symptoms and conditions such as irritable bowel syndrome is higher (~19%) in these patients over healthy individuals [125]. This suggests that the microbiome and diet might have a role in the immunopathology of schizophrenia.

#### 4.2.2. Drugs

Antipsychotic drugs or neuroleptics have both intended (therapeutic) and unintended (adverse/side effects) effects. For instance, the antipsychotic drug clozapine’s main target is dopamine and serotonin receptors, and this interaction leads to the alleviation of positive and negative symptoms in many patients. In addition, it acts partially on muscarinic, adrenergic, and histamine receptors, which leads to side effects such as drowsiness and dizziness [126]. Similarly, there is a vast literature that implicates antipsychotic medication as having another function, which is to alter the cytokine network in the schizophrenia population [127,128,129,130]. It is not yet understood how this medication alters cytokine levels, but we can hypothesise two possibilities: Firstly, they could affect the cytokine levels directly by unknown means, or they alter the TLR expression/protein levels and regulators in their downstream signalling, further influencing the cytokine expression. It could possibly be the latter, as TLRs regulate cytokine expression. In congruence with this statement, a study showed that paliperidone [131] and clozapine [132] reduced the inflammatory response (LPS-induced) associated with TLR4 by partly inhibiting the calcium/calmodulin-dependent Akt (protein kinase B) in a rat microglial cell line. Inhibition of this enzyme prevents Akt-dependent IKKα phosphorylation, further preventing NF-kB nuclear translocation [132]. Moreover, the MIA model of mice using poly I:C reported that paliperidone prevented the upregulation of TLR3 protein in the frontal cortex region [133]. On the contrary, another antipsychotic treatment, olanzapine, resulted in a significant rapid elevation of inflammatory cytokines (IL-6, TNF-α, IL-1β) and TLR4 protein expression in rat PFC [134]. A study on unmedicated patients reported significantly higher expression (MFI) of TLR2 in monocytes post-antipsychotics treatment, whereas TLR4 and 5 MFI were significantly higher before medication, and no difference after [70]. These studies indicate that drugs alter TLR downstream signalling, which leads to cytokine imbalance. On the other hand, studies reported that antipsychotic-treated patients showed higher expression of *tlr4* mRNA [66,135] and *myd88* mRNA [66] in peripheral blood and postmortem brain tissue samples, respectively, over antipsychotic-free patients (schizophrenia). Interestingly, no significant differences were observed in the other downstream signalling protein mRNA levels such as *nf-kb*, *ikbα*, *il-1β*, and *il-6* [66]. On the other hand, 3 months of antipsychotic treatment resulted in higher TLR6 protein expression, and a trend towards higher TLR8 and 9 in monocytes, T cells, and B cells of FEP patients [63]. Given the evidence, there is a disparity in the current literature regarding the influence and relationship between antipsychotic drugs and TLRs and cytokines which is not yet clearly understood, and studies are ongoing to find the same.

Studies have reported that recreational drugs such as alcohol consumption (binge) and frequent or continuous use of amphetamines and methamphetamines induces psychosis-like symptoms in nearly half the users [2,136]. It was found that methamphetamine elevated IL-1β and IL-18 in primary mouse astrocyte cultures, and this was mediated by TLR4, NF-kB, and caspase-11 pathways, causing neuroinflammation [137]. This is the most probable reason why most of these users are at significant risk of schizophrenia where some of them develop psychosis, which eventually transforms into schizophrenia [138].

#### 4.2.3. Genetics

Genetic polymorphisms represent DNA variation in a specific population that may/may not lead to a change in protein expression. One of the commonly occurring polymorphisms is a single nucleotide polymorphism (SNP), which has been used as a genetic target to study multiple diseases [139]. SNPs within multiple immune-related gene networks have now been investigated in terms of their association with disease risk. Among these, it was found that polymorphisms within genes encoding *tlrs* and their pathways were associated with either increased or decreased protection against various diseases and infections. Moreover, altered expression of cytokines in response to infections was associated with *tlr* gene polymorphisms [140].

In schizophrenia, gene polymorphisms associated with pathways of inflammation, immune response, neurodevelopment, and cell death have been identified. Alterations of such pathways due to polymorphisms are significantly associated with schizophrenia risk, and the TLR pathway is suggested to be a modulator of these pathways [141]. Studies also show that TLR polymorphisms are associated with schizophrenia (Table 3), such as *tlr2* polymorphism in the Tunisian population [142]. An insertion/deletion polymorphism of an SNP (rs111200466) located in the promoter region of the *tlr2* gene showed to be protective in healthy females, as this polymorphism was not observed in the schizophrenia group [142]. Missense polymorphisms (rs5743708 and rs121917864) located in the third exon of *tlr2* gene were considered to increase the risk and susceptibility to schizophrenia. The aforementioned three SNPs were previously associated with decreased activation and expression of TLR2 and cytokines [142]. In another study, two SNPs, rs3804099 and rs3804100 (of unknown function), located in the third exon of *tlr2* gene, were reported to be significantly associated with poor concentration in the Korean schizophrenia population. Although these 2 SNPs were found to be associated with cognition, none were associated with either positive or negative symptoms [143]. Patients belonging to north Indian ethnicity showed a similar association with *tlr2* SNP (*rs3804099*) [144]. This indicates the broad (multiethnic) implications of TLR2 polymorphism in schizophrenia. TLR4 polymorphism also showed an association with schizophrenia risk; four SNPs of TLR4 were investigated, of which three (rs11536889-3′UTR, rs1927911-intron, and rs1927914-5′UTR) showed an association with schizophrenia. One of these investigated SNPs was previously associated with multiple diseases and disorders [145]. Changes in the TLR downstream signalling proteins such as *myd88* SNP (rs7744-3′UTR) were reported to be less prominent in patients with schizophrenia than in controls. Moreover, a model utilising 5 SNPs (3 TLR downstream signalling protein genes (*myd88*, *irak1*, and *nfkb1*) and 2 cytokines (*il6* and *il1b*)) for a five-way analysis identifies different combinations of these five genes, predicting the risk of schizophrenia in a given population. This model showed that people having a specific combination of these five genes were at higher risk (OR—6.9) of developing schizophrenia [66]. This evidence indicates the immune–genetic predisposition in schizophrenia, and it can be understood that multiple polymorphisms of various genes associated with the TLR pathway collectively contribute towards the incidence of schizophrenia (Table 3).

## 5. TLRs in Cognitive Impairment in Schizophrenia

Cognitive deficits are widely reported in schizophrenia and are strongly predictive of social and occupational function. This includes impairment in the domains of episodic and working memory, executive function, attention, and social cognition, ultimately resulting in poor quality of life (QoL) [146]. Poor QoL has been identified as one of the contributing factors towards suicidal ideation [147], and in schizophrenia, suicidal ideation was found to be approximately 13 times more common in comparison to healthy individuals [59]. This suicidal ideation was, in turn, greatly associated with cognitive deficits [148]. There is a vast amount of literature showcasing cognitive deficits as a core aspect of this disorder, but the root of its dysfunction is not yet known.

It has been widely speculated that neuroinflammation might be involved in the development of cognitive deficits. We found in our recently published meta-analysis that peripheral inflammatory markers (IL-6, IL-1β, TNF-α, and C-Reactive protein) showed an association with cognitive impairment [149]. Although it is not yet known to what extent these cytokines, chemokines, and interferons individually/collectively contribute towards cognitive decline, TLRs have been implicated many times in cognitive impairment, whether it was bacterial infections or other neurodegenerative diseases such as Alzheimer’s and Parkinson’s disease, and psychiatric diseases such as schizophrenia [9,97,150]. Hence, it could be a good approach to understanding cognition from a TLR perspective.

Studies performed either by knocking out TLR genes or by administering agents inhibiting/stimulating its activity showed that several TLRs, including *tlr2* [150,151], *tlr3* [98], and *tlr4* [96,101] were found to be associated with cognitive dysfunction (Table 2). Among these, TLR2 expression is thought to be strongly associated with cognitive impairment with either prenatal or postnatal stimuli. Prenatal TLR2 stimulation in dams shows decreased social interaction and affected contextual memory, impairing cognitive performance in their female offspring [95]. Whereas, postnatal TLR2-mediated neuroinflammation shows impairment of spatial and fear-comprehending cognitive functions [40]. The presence of TLR2 is believed to be essential for regular cognitive function, as knocking out TLR2 (TLR2^−/−^) in mice caused enlargement of ventricles, and behavioural and cognitive impairment [150]. It was also observed that such mice (TLR2^−/−^) exhibited anxiety-like behaviour [152]. It could likely be due to sensorimotor gating dysfunction and other molecular changes (Akt dysregulation) mimicking schizophrenia-like symptoms, but antipsychotic intervention effectively mitigated this dysfunction [150]. Furthermore, in such mice, providing training repeatedly improved their learning ability and memory [153], suggesting molecular pathways other than TLR2 are involved in cognitive deficits. Comparably in humans, antipsychotic drugs increased TLR2 (MFI) expression in monocytes of patients with schizophrenia, indicating a possible association between TLR2 and cognitive performance by which antipsychotic medication ameliorates cognitive impairment [70]. However, the exact connection between elevated TLR2 protein expression and improvement in cognitive performance is not known. On the contrary, decreased cognitive performance in patients with schizophrenia was observed with higher TLR2 activity (whole blood-stimulated) and elevated IL-6 plasma. These elevated IL-6 levels were further shown to mediate the association between childhood trauma and emotion recognition deficits [107]. This further indicates that inflammation and TLRs affect cognitive performance in schizophrenia.

Although TLR2 is shown to play a role in cognition, it would be incorrect to assume that it is the only pathway that might be involved in cognitive deficits in schizophrenia; other pathways or other TLRs such as TLR3 and TLR4 are involved with cognitive performance as well. Poly I:C treatment in dams led to TLR3-dependent defective cerebral cortical proliferation and neurogenesis in the foetus, causing behavioural abnormalities later in the offspring [51]. TLR4+ monocytes in peripheral human blood (FEP) were found to be positively associated with visual learning and working memory [154]. However, these results were inconsistent with evidence from Keri et al., that a decrease in cognitive functions was associated with an increase in TLR4+ and TLR5+ monocytes in the FEP group [70]. These contrasting results may reflect TLR4’s dual role (increasing/decreasing) in neuroplasticity, neurogenesis, and cognitive functions [154], or its complex interaction with other factors (TLR downstream signalling, medication, individual monocytes/immune cells combined). Antagonising TLR4 (intraperitoneal administration of antagonist) improved spatial memory in young female mice [96]. Although the effect observed is opposite to that of TLR2, it indicates that other TLRs might be at play. A study that investigated the role of monocytic TLR4 signalling in cognitive deficits in schizophrenia groups (tardive dyskinesia and nontardive dyskinesia) showed that high TLR4 protein expression (MFI in the monocytes) was associated with lowered reasoning, problem-solving, and overall cognitive performance [72], and high mRNA expression [71], with a decline in social cognition and processing speed in patients with schizophrenia. This effect was more pronounced in the schizophrenia group with tardive dyskinesia [72]. Other proteins such as IFITM3 (interferon-induced transmembrane protein 3) gene expression was elevated in animals treated with poly I:C and LPS. This protein plays an active role in antiviral responses, and was reported to be involved in regulating humoral and growth factors involved in immune response and neurite elongation, ultimately affecting neurodevelopment [155] and resulting in impairment in memory and social behaviour, indicating the deleterious effects upon TLR3 and 4 activations on cognition [98,100]. This *ifitm3* gene in dorsolateral prefrontal cortex samples was previously reported as one of many upregulated genes in patients with schizophrenia [156]. Another protein, CaMKIIα (calcium-calmodulin dependent kinase II alpha), activity in the hippocampus was decreased with upregulation of TLR4/MAPK/ERK activity in mice. This is an enzyme which was previously associated with behavioural and synaptic regulation in a mouse schizophrenia model. In this model, the knockdown of TLR4 rescued mice from CaMKIIα depletion, further improving social cognition and behaviour [157].

Cognition is also influenced by other TLR signalling cascade proteins. SARM1 (sterile-α and armadillo-motif-containing protein 1) is one such mammalian protein, which is a TRIF inhibitor in the TLR pathway [158]. Animal studies show that this protein is neuron-specific and absent in glial cells [159]. SARM1 knockout studies in mice revealed that the absence of this protein impaired memory, both general and social cognitive ability, and behaviour [160]. Furthermore, this protein regulates the cytokine expression in the brain [159], indicating that downregulation/impairment of this protein may upregulate TLR downstream signalling, leading to cytokine imbalance, and further impairing cognitive functions. Another protein, MyD88 was found to affect the behavioural and cognitive performances in mice. The absence of this MyD88-dependent TLR pathway led to poorer performances in working memory [97]. Studies also found that activation of TLR7 and IL-6 restricted neuronal dendrite growth, further negatively affecting their exploratory behaviours [99]. This indicates the crucial role played by TLRs and their proteins in cognitive function, further suggesting the importance of TLRs (neuroinflammation) in schizophrenia. Given, where our knowledge currently stands, we can only hypothesise about a possible connection between TLRs and cognitive impairment in schizophrenia. In addition to the TRIPS (Toll-like receptors in immuno-inflammatory pathogenesis) theory [161], which proposes that schizophrenia risk is associated with neurodegeneration mediated by TLR3 and TLR4 activation, we hypothesise (Figure 1) that stressful events during the neurodevelopmental stage, either due to MIA, childhood trauma, or other insult, results in increased ROS (reactive oxygen species) followed by damage to the cells in the brain, which in turn produces DAMPs and cytokines that are recognised by TLRs on microglia, causing microglial activation. The activated microglia release cytokines as a response, suppressing the neuronal development, causing cognitive impairment. In addition, various other factors play a role, such as genetic polymorphisms, TLR signalling proteins and their regulators, and aforementioned proteins (such as IFITM3 and CaMKIIα) that are directly/indirectly affected by the altered TLR signalling (Figure 1).

## 6. Conclusions and Future Perspectives

Toll-like receptors have previously been implicated in various neurodegenerative and psychiatric disorders. Their role in the neuroinflammatory response and their association with cognition has always been in question. In this current review, we showcase some of the many important features of TLRs: their altered expression and activity in schizophrenia, factors affecting TLR expression, and their significance in altered cognitive performance. We also showcase the role of TLR2, 3, and 4 in schizophrenia, as they are extensively studied compared to the rest of the TLRs. However, the evidence connecting TLRs to cognitive deficits in schizophrenia is still lacking, as there are only a few animal studies, and even fewer in humans. Despite the lack of evidence on the pathways connecting both of them, there is a growing interest in exploring their association and the impact of TLRs on other signalling proteins affecting cognitive functions. It is evident from animal and human studies that TLRs are one of the many pathways that may lead to cognitive impairment. Both lower and higher expression of TLRs contribute towards cognitive deficits. Hence, normal TLR functioning is essential for regular cognitive performance, and has a substantial role in the immunopathology of schizophrenia.

There is much conflicting evidence in the literature regarding TLR activity in schizophrenia, as the evidence presented is from various parts of the body such as brain tissue, blood, and its components, which informs us that TLRs have different behaviour in patients with schizophrenia. Their altered activity and expression could either be due to a compensatory mechanism [68], polymorphism, direct insult, or other unknown underlying reasons. We have highlighted a number of factors that can influence TLR expression and activity, such as drugs and the microbiota, but these factors can change with time, and the temporal changes in TLR activity and expression in schizophrenia are currently unknown. TLR expression and immune responses in general are dynamic in nature, and studies reporting measurements at single time points fail to capture changes over longer periods of time, and even diurnal variations. It is also important to state that TLR expression and activity may vary with the phase of the illness. It may also change with metabolic activity, hormonal variation, ageing, and with the introduction of newer medications for comorbid illnesses.

Although the reason is unclear, at present we can only speculate on the connection between TLRs and cognitive deficits in schizophrenia (Figure 1), and presume that elevated cytokines due to altered TLR activity because of insults during neurodevelopment age lead to suppression of neuronal growth and synaptic strength. However, it is not yet clearly understood why there is an altered cytokine and TLR expression in patients with schizophrenia. Understandably, adverse biological events in foetal or childhood, and experiencing the first episode of psychosis can elevate cytokines, but the reason for long-term maintenance of these abnormal levels of cytokines and TLR expression in these patients is under question. Exploring the signalling pathways of TLRs in regions of the brain associated with cognition, and their impact on microglial activation and neurodegeneration, is essential. Measuring both TLR and cytokine expression in peripheral measures is crucial, and could enable us to understand the issue under question. Understanding this could increase the potential targets and pharmacological strategies to alleviate cognitive impairment in schizophrenia, and improve overall QoL in these patients.

## Figures and Tables

**Table 1:** Characteristics of human studies.

Study	Diagnosis of Schizophrenia	Sample Type	TLR Analysed	Analysis Result
**García-Bueno et al., 2016** [66]	DSM-IV. Absence of other neuropsychiatric disorders and drug abuse.	Postmortem human brain tissue (PFC).	TLR4 and MyD88 protein and mRNA expression.	Significant higher TLR4, MyD88 not IkBa and NFkB protein expression in patients. No change in MyD88 mRNA expression. Significant higher TLR4, MyD88 not IkBa protein expression and MyD88 mRNA expression in patients treated with antipsychotics vs. controls. Significant higher NFkB protein expression in antipsychotic free patients vs. controls.
**López-González et al., 2019** [65]	DSM-IV and ICD-10. No history of neurological diseases.	Postmortem humanbrain tissue (PFC).	TLR4 and TLR7 mRNA expression.	Significant downregulation of TLR4 not TLR7 in patients.
**MacDowell et al., 2017** [64]	DSM-IV and ICD-10. No history of neurological diseases.	Postmortem humanbrain tissue (PFC and CB).	TLR4 and MyD88 protein expression.	Significant lower TLR4, MyD88, and IkBa, not NFkB in patients (PFC). Significant higher TLR4, MyD88, and IkBa, not NFkB in patients (CB).
**Chase et al., 2019** [62]	SCID-IV. No current or previous psychiatric history.	PBMC.	TLR4 mRNA expression.	Adverse childhood events were associated with IL-6 expression, but not TLR4.
**Juncal-Ruiz et al., 2020** [63]	DSM-IV. Absence of other neuropsychiatric disorders and drug abuse.	PBMC.	TLR1–9 protein expression.	FEP patients showed significantly higher TLR1, but not TLR 5 and 8 in PBMCs. Three months of antipsychotic treatment led to increase in TLR6 monocyte expression, and a similar trend for TLR8 and 9 in PBMCs.
**Kozłowska et al., 2019** [61]	DSM-IV and ICD-10. No history of neurological diseases.	PBMC.	TLR1–9 mRNA.	TLR1, 2, 4, 6, and 9 expressions were downregulated in patients. TLR3 and 7 expressions were upregulated in patients. TLR5 and 8 no change.
**Chang et al., 2011** [67]	Patients were recruited if they experienced psychosis (hallucinations or delusions).	Monocytes.	TLR1–6 and 9 mRNA expression.	Downregulation of TLR3 and 5 mRNA in patients. No change with other TLRs.
**Kéri et al., 2017** [70]	SCID-CV. No current or previous psychiatric history.	Monocytes.	TLR1, 2, 4, 5, and 6 protein expression.	Significant higher TLR4 and 5 expression in patients. At baseline, TLR4 and 5 expression correlates with RBANS scores, but not postmedication.
**Li et al., 2022** [71]	DSM-IV. Absence of other neuropsychiatric disorders and drug abuse.	Monocytes.	TLR4 protein expression.	Elevated TLR4 was associated with decreased processing speed and social cognition.
**Li et al., 2022** [72]	DSM-IV. Absence of other neuropsychiatric disorders and drug abuse.	Monocytes.	TLR4 protein expression.	Elevated TLR4 was associated with decreased reasoning/problem-solving and MCCB scores in schizophrenia patients with tardive dyskinesia, but not with no tardive dyskinesia or controls.
**Müller et al., 2012** [68]	DSM-IV.	Monocytes stimulated with poly (IC), LPS.	TLR2, 3, and 4 protein expression.	No change with TLR2. Significant higher TLR3 in patients vs. controls (unstimulated and PolyIC); no changes in LPS stimulated. Significant higher TLR4 in patients vs. controls (unstimulated); no changes in LPS and polyIC stimulated.

Characteristics of Human studies illustrating TLRs in various models and cognition. PFC—prefrontal cortex, CB—cerebellum, PBMC—peripheral blood mononuclear cells, LPS—lipopolysaccharide, TLR—Toll-like receptor, RBANS—Repeatable Battery for the Assessment of Neuropsychological Status, MCCB—Measurement and Treatment Research to Improve Cognition in Schizophrenia (MATRICS™) Consensus Cognitive Battery, MyD88—myeloid differentiation primary response 88, NFkB—nuclear factor kappa B, IkBa—inhibitor of nuclear factor kappa B alpha, poly (IC)—polyinosinic:polycytidylic acid, LPS—lipopolysaccharide, DSM-IV—Diagnostic and Statistical Manual of Mental Disorders-IV, SCID-CV—Structured Clinical Interview for DSM-Clinical version, ICD-10—International Classification of Diseases-10.

**Table 2:** Characteristics of animal studies.

Study	Animal (Type of Cells/Culture)	Model	TLR Analysed	Analysis Results
**Talukdar et al., 2021** [93]	Rats	MIA (Poly IC and LPS)	TLR2, 3, and 4 mRNA	Elevated TLR genes were highly associated with a decrease in sociability and normal behaviour in the offspring.
**Lee et al., 2016** [95]	Mice	MIA (Zymosan)	TLR2	TLR2 stimulation showed decreased cognitive and behavioural changes only in female offspring.
**O’Loughlin et al., 2017** [94]	Mice	MIA (LPS)	TLR2 and 4 mRNA	Elevated TLR genes in pre/postnatal stages after stimulation. These were associated with morphological changes in microglia, suggesting activation.
**Connolly et al., 2021** [96]	Mice	TLR 4 antagonism	TLR4	TLR4 antagonism showed beneficial cognitive effects (enhanced spatial learning and memory) only in young female mice.
**Drouin-Ouellet** ** et al., 2012** [97]	Mice	MyD88 KO	TLR	The absence of MyD88 in mice led to spatial learning and working memory deficits, and reduced motor activity.
**Ibi et al., 2009** [98]	Mice	Poly IC stimulation	TLR3	TLR3 stimulation showed decreased social interaction, cognitive, and behavioural changes.
**Liu et al., 2013** [99]	Mice	TLR7 KO	TLR7	TLR 7 activation reduced dendrite growth mediated by IL-6 in mice. TLR 7 KO reduced dendrite growth and reduced behavioural activity.
**Madar et al., 2015**	Mice	TLR2 KO and TLR2+	TLR2	TLR2 KO decreased mice activity and impaired spatial learning. Neonatal TLR2/1 activation led to impaired spatial learning in adulthood.
**Park et al., 2015**	Mice	TLR2 KO	TLR2	TLR2 KO mice showed cognitive and social recognition impairment.
**Melnik et al., 2012**	Mice	In utero (poly IC stimulation)	TLR3 mRNA	TLR3 stimulation leads to decreased proliferation after Poly IC treatment unlike, in vitro studies.
	Mice (DG NPC)	Poly IC treatment	TLR3 mRNA	TLR3 stimulation leads to increased proliferation after 24 and 48 h of poly IC treatment.
**Liu et al., 2013** [99]	Mice (cortical neurons)		TLR 7	TLR7 activation produced cytokines and hindered dendrite growth. TLR7 KO promoted axon and dendrite growth.
**Nakajima et al., 2014** [100]	Mice (astrocyte cell culture)	LPS stimulated	TLR3 and 4 mRNA	LPS stimulation showed significantly elevated TLR3 expression up to 24 h and dropped until 72 h. No significant change was seen with TLR4 expression, but a trend of upregulation was observed from 3 to 72 h.
**Zhou et al., 2020** [101]	Mice (BV2 microglial cells)	LPS treatment	TLR4 protein	TLR4 and NF-kB upregulation post-treatment.

Characteristics of animal studies showcasing the effect on TLRs in various models and cognition. DG-NPC—Dentate gyrus-neural progenitor cells, LPS—lipopolysaccharide; TLR—Toll-like receptor, MIA—maternal immune activation, KO—knockout, IL-6—interleukin-6.

**Table 3:** Characteristics of genetic studies.

Study	Population	Gene (SNPs)	Location of SNP	Region of SNP	SNP Function	Analysis Results
**Aflouk et al., 2021** [142]	Tunisian	*tlr2* (rs111200466)	Chr4. −196 to −174 Insertion/Deletion	Promoter region	Decreased activation and expression of TLR2 and cytokines	Protective action in females, as this polymorphism was not observed in the schizophrenia group (females).
		*tlr2* (rs5743708)	Chr4. G > A Missense variant	Third exon (functional)		Increases the risk and susceptibility to schizophrenia.
		*tlr2* (rs121917864)	Chr4. C > T Missense variant			
**Kang et al., 2013** [143]	Korean	*tlr2* (rs3804099)	Chr4. T > C Synonymous Variant	Third exon (functional)	Unknown function	C’ allele associated with cognition (poor concentration).
		*tlr2* (rs3804100)	Chr4. T > A/T > C Missense variant			
**Sharma et al., 2022** [144]	Indian	*tlr2* (rs3804099)	Chr4. T > C Synonymous Variant	Third exon (functional)	Unknown function	Increases the risk of schizophrenia.
**Mostafa et al., 2022** [145]	Egyptian	*tlr4* (rs11536889)	Chr9. G > A/G > C UTR Variant	3′UTR	Interferes with the translation process and increase the expression of TLR4	A strong association between these genes and schizophrenia.
		*tlr4* (rs1927911)	Chr9. A > C/G/T	Intron	-	
		*tlr4* (rs1927914)	Chr9. G > A	5′UTR	Regulation of gene expression and protein levels	
**García-Bueno et al., 2016** [66]	Spanish	*myd88* (rs7744)	Chr3. A > G/A > T Noncoding Transcript Variant	3′UTR	Previously associated with autoimmune or inflammatory processes	These two polymorphisms were found to be crucial in the schizophrenia predictive model for this population.
		*il6* (rs1800795)	Chr7. C > G/C > T	Promoter region	Increases IL-6 levels	

Characteristics of genetic studies illustrating polymorphisms in schizophrenia group in various populations. Chr—chromosome, A/G/T/C—nucleotide bases (adenine (A), cytosine (C), guanine (G), and thymine (T)), tlr—Toll-like receptor, il6—interleukin-6, myd88—myeloid differentiation primary response 88 protein, rsxxxxxx—reference SNP “ID”, SNP—single nucleotide polymorphism, rs—reference SNP, UTR—untranslated region.

## Data Availability

Not applicable.
